# A Comparative Study of Density of Obturated Root Canals by Conventional and Mechanical Lateral Condensation Techniques

**Published:** 2009

**Authors:** Hasan Abedi, Shahriar Shahriari, Seyed Mohsen Jalalzadeh, Reza Moradkhany

**Affiliations:** * Assistant Professor, Department of Endodontics, School of Dentistry, Hamadan University of Medical Sciences, Hamadan, Iran; ** Private Practice Dentist, Hamadan, Iran

**Keywords:** Dental high-speed equipment, Gutta-percha, Root canal obturation

## Abstract

**Background:**

The purpose of this study was to compare the weight of root canal filling material used in a new lateral condensation technique named mechanical lateral condensation (MLC) with that of conventional lateral condensation technique (LC). This new technique uses reciprocal handpiece.

**Methods:**

In this experimental study, 40 human extracted straight canine teeth were used. After crown amputation, root canals were prepared with 18 mm in length. The teeth were randomly divided into four experimental groups; each group was consisting of 10 teeth. The teeth in groups IA and IB were filled by LC technique. The teeth in groups IIA and IIB were filled by MLC technique and using a finger spreader that placed in a reciprocating-action handpiece to laterally condense cold gutta-percha, creating space for accessory cones. All of the roots were weighted before and after obturation and the difference demonstrated the weight of gutta-percha mass. The data were analyzed by t-test.

**Results:**

The mean weight for MLC obturations was 10.82 ± 0.025 g compared with 7.37 ± 0.035 g for that of LC technique. The difference was statistically significant (P = 0.001).

**Conclusion:**

This study showed that MLC technique requires more gutta-percha mass than LC technique.

## Introduction

The goal of root canal filling is to completely obliterate the canal space with a stable, nontoxic material whilst at the same time creating a hermetic seal to prevent the movement of tissue fluids, bacteria or bacterial by-products through the filled canal. To date the material most used in endodontics is gutta-percha in combination with a root canal sealer. The sealer provides the seal, not the gutta-percha, yet it has been reported that some sealers shrink upon setting whilst others are susceptible to breakdown.^1^,^2^ Therefore, the amount of sealer should be kept to a minimum and only be found in a thin layer between gutta-percha and the wall of the canal.^3^ To accomplish this task, the amount of gutta-percha packed into the canal must be maximized. Currently, the two most popular gutta-percha obturation techniques are cold lateral condensation (LC) and warm vertical condensation (WVC). LC is the obturation technique most widely taught in dental schools, and used by practitioners, and is still the standard to which all other techniques are compared.^4^ However, the technique can result in the creation of void, spreader tracts, excessive amounts of sealer, and lack of surface adaptation to canal walls.^5^,^6^ Alternative techniques, which incorporate the use of thermal or frictional heat to plasticize the gutta-percha, allowing better adaptation to canal walls and a higher degree of homogeneity, have been introduced.^7^ One of these methods, mechanical lateral condensation,^8^ involves placing a master cone in the canal, followed by a nickel-titanium spreader activated by a reciprocating-action handpiece.

The aim of this study was to compare the weight of gutta-percha mass used in LC technique and that of the mechanical lateral condensation (MLC).

## Materials and Methods

In this experimental study, forty extracted human straight canine teeth, with fully formed apices were used. Teeth were immersed in 2.5% NaCLO for 2 hours and stored in normal saline. After crown amputation with a high speed handpiece and a carbide fissure e bur (Tees Kavan co, Tehran, Iran), the canals, 18 mm in length, were enlarged to a size 35 master apical file with K-file (Mani, Tochigi, Japan) and flared to size 80 using the stepback instrumentation technique. Irrigation was performed with 2 ml normal saline by a 27-gauge needle (Supa, Tehran, Iran), between each instrumentation. Apical patency was performed by a size 10 K-file (Mani, Tochigi, Japan). Following the complete instrumentation, the canals were irrigated with 17% EDTA (MD cleanser, Meta Biomed co, Chong Ju City, Korea) and 5.25% sodium hypochlorite to remove smear layer.^9^ Teeth were randomly divided into four experimental groups. After canal preparation, all of the roots were weighted to the nearest 0.001 g using a digital scale (Sartorius, Gottingen, Germany). In the four experimental groups, one-half of the obturations were accomplished using the LC technique and one-half were done using the MLC technique. For the LC technique, a size 35 gutta-percha master cone (Aria, Tehran, Iran) was placed to the working length. Then, a medium-fine Ni-Ti finger spreader (Hygienic Crop., Chicago, IL) was advanced into the canal until the resistance occurred. The spreader was rotated in a reciprocating motion with apical pressure, attempting to penetrate it to within 1 mm of the working length. After the apical progression of spreader stopped, apical pressure was maintained for about 60 seconds, and then the spreader was removed. A size 20 gutta-percha accessory cone (Aria, Tehran, Iran) was inserted into the created space in the canal and advanced apically as far as possible. The process was repeated until the spreader could not penetrate into the coronal one-third of the canal length. The other half of the obturations was accomplished using the MLC technique. With the same canals, spreaders, and materials that were used in the LC technique. But the spreader was seated into the head of the reciprocating handpiece NSK (TEP-E10r, Nakanish Inc., Tokyo, Japan). A rubber stopper was placed on the spreader at the point of 1 mm shorter than the working length and the spreader was inserted into the canal alongside the master cone until the resistance was felt. The handpiece was set at the maximum speed setting. The handpiece was activated and a light force was applied to advance the spreader slowly in apical direction to the desired or maximum level of the penetration. Activation was continued at this level for about 1-5 seconds and during removal of the spreader. Gutta-percha accessory cones were placed and obturation was completed using the same procedures that were used with traditional LC.^8^

The following four experimental groups were created: group IA, 10 obturations completed by operator A using the LC technique; group IB, 10 obturations completed by operator B using the LC technique; group IIA, 10 obturations completed by operator A using the MLC technique; group IIB, 10 obturations completed by operator B using the MLC technique.

The two operators obturated the canals either using the LC or MLC technique, with no sealer. After obturation, gutta-percha cones were cut with blade at the orifice of the canals. Then, the roots were weighted again. The difference between the weight of the roots before and after obturations demonstrated the weight of gutta-percha mass. The data were analyzed with t-test.

## Results

The mean weight for all MLC obturations was 10.82 ± 0.025 g compared with 7.37 ± 0.035 g for LC. The difference was statistically significant (P = 0.001).

The difference between the two operators in groups IA and IB and IIA and IIB was not statistically significant (P = 0.08).

## Discussion

In previous studies, one method of evaluating the quality of root canal obturations has been the visual inspection of the obturation material, either with the aid of a microscope or by radiographs.^10^–^12^ Due to the subjective nature of these examinations, a more objective quantitative method was designed to compare different obturation techniques. Liewehr et al^13^ used clear plastic blocks with embedded straight canals commonly used as endodontic teaching aids. They weighted the blocks after standard cold lateral condensation and after lateral condensation technique using Endotec thermal endodontic condenser. The density or weights per volume unit of the gutta-percha from the two methods were compared. An increase in weight of the filling material in the same volume (i.e., the instrumented canal) implied an increase in the density of the obturation and therefore, a denser, complete root canal filling maximize the amount of core material, as advocated by Schilder.^7^ In the present study, several changes were made to update the technique and to simulate a more realistic clinical situation. First, canine root canals were used in this study because they are long, relatively straight and are usually oval, offering a better test of the ability of a technique to fill irregularities, compared with roots with round canals. Second, the canals were instrumented to a file size of 35 at the apex, and flared to size of 80 using the stepback instrumentation technique. Third, the same spreaders and materials were used in all experimental groups.

In similar researches, other methods of compacting gutta-percha have also produced greater weights of fills than LC weights. Liewehr et al^13^ compared density of gutta-percha in lateral condensation and warm lateral condensation. There was a 14.6% more increase in weight after warm LC. Wong et al used split molds, so the same canal could be used for repeated obturation. They demonstrated that obturations using vertical condensation were 2.8% heavier than those with LC.^14^ Gound et al^8^ compared the weight of gutta-percha used in obturation between the LC and the MLC techniques in resin blocks. The MLC technique obturations were significantly heavier on average than those of the LC fills. In another research, Gound et al compared the effect of spreader and accessory cone size on density of obturation using conventional or mechanical lateral condensation techniques. The MLC technique fills were significantly heavier and had greater depth of penetration on average than those of the conventional lateral condensation.^15^ The best combination for heavy fills was the MLC, fine-medium spreaders, and fine accessory cones. The greatest mean accessory cone depth occurred with the MLC, fine-medium spreaders, and size 25 accessory cones.^15^ Jarrett et al compared the apical density of several obturation techniques when used in palatal roots of extracted maxillary molars. Results recommended that Thermafil, mechanical lateral and WVC (Schilder) obturation techniques created more complete obturation using gutta-percha at 2 and 4 mm levels compared with cold lateral, WVC (continuous wave).^16^

## Conclusion

In the present study, MLC obturations were significantly heavier than LC obturations. With regard to results of these studies, it seems that the MLC technique is a greater and suitable method for root canal obturation. However, complemetary studies such as evaluation of the apical microleakage and penetrating depth of spreader are recomended.
